# Supplementary material for: Analytical Studies of Antimicrobial Peptides as Diagnostic Biomarkers for the Detection of Bacterial and Viral Pneumonia
Source: Bioengineering (Basel). 2022 Jul 11;9(7):305. doi: 10.3390/bioengineering9070305 (PMC9311714; doi:10.3390/bioengineering9070305)
Supplement: Supplementary file 1 [file bioengineering-09-00305-s001.zip › bioengineering-1763051-supplementary.pdf]

Putative AMPs

BOPAM-AB

1 FLPIVGKLLKSGLSGLL

2 FFPIVGKLLKSGLSGLL

3 FFPIVGKLLKFGLFGLL

4 FFPIVGKLLKFGLSGLL

5 AIKTVGKAVGKGLRAIN

BOPAM-KP

1 VCKEEGRSSGHCSPSLKCWCEGC

2 MWKYVVKGGLNNGVQCKIDNC

3 MWKHVVKLGNNFTVQCKIDNC

4 IHHEAGKGGYIPYLKWHLRKK

5 ICKEEGRISGHCSASLKCWFKKR

6 LAKKAKKAGAEKKKCKELAKK

7 LCREEGHVSGHCSASLKCWRAMK

BOPAM-SP

1 QGRDDRYCESIMRRRGLTSPCKDINTFIHGNKRSIKAICENKNG

2 KGRNDRYCESMMERRGLTTPCKDTNTFIHGNKGSIKAICGNKNG

3 FRHDSGIGEYEVHHQKLVFFAEDVSGSNKGFCAIIGLMVGGVVI

4 FRHDSGIGEYEVHHQKLVFFAEDVSGSNKGFCAIIGLMVGGIVI

5 FRHESGIGEYEVHHQKLVFFAEDVSGSNKGFCAIIGLMVGGVVI

6 FRHDSGIGEYEVHHQKLVFFAEDVSGSNKGFCAIIGLMVGGVVI

7 YRHDAAIGEYEVHHQKLVFFAEEVSGSNKGFCAIIGLMVGGVVI

8 KGRDNKYCETMMEKRHLTKPCKSINTFVHGNKNDIKDICKDKNG

INFLUENZA A

1 TPTFIDGQVPIPKQ

2 CPVILDSAIQVLPK

3 TPTFIDGQVTVPDQ

4 TPTFIDGQVPMPQQ

5 TPTFIDGQVPIPQQ

6 TPKFIDGQVPIPEQ

7 CPVILDSSIQVFPK

8 TPTFIDGQVPIPEQ

INFLUENZA B

1 MDVSHRWTFLRVPPQ

2 MDVSHRWTFLMVPPQ

3 LNCNPQLLCLNCNPQ

4 LKILQLLLFLKVPQL

5 LTILQLLLFLKVPQL

6 LNCNPPLLCLNCNPQ

RESPIRATORY SYNCYTIAL VIRUS

1 IVSSIKEEINLCKNKF

2 EVSKINEKIDSNLSTV

3 NIVDVNKKIDANTTAI

4 KVTEINANIDNNVNII

5 AINSVSAQVNKNTNNI

| | |
|---|---|
| 6 | KFSQICEAIDMQTSVI |
| 7 | NISNVKNELNQNINNV |
| 8 | SIYNFNENSNVLLSAV |
| 9 | NISKVTNQVNTNTTNI |
| 10 | VINDVSKQVNTNTTNI |
| 11 | NVTNISNKVDVNTADI |
| 12 | DITNINNNIDAKFTKI |
| 13 | KIRDLNEKLDDRITNV |
